# Forest fragmentation causes an isolated population of the golden takin (*Budorcas taxicolor bedfordi* Thomas, 1911) (Artiodactyla: Bovidae) in the Qinling Mountains (China)

**DOI:** 10.1186/s40850-024-00192-1

**Published:** 2024-01-30

**Authors:** Hui Feng, Fangjun Cao, Tiezhi Jin, Lu Wang

**Affiliations:** Shaanxi Key Laboratory of Qinling Ecological Security, Shaanxi Institute of Zoology, 710032 Xi’an, China

**Keywords:** Microsatellites DNA, Population structure, Population bottlenecks, Conservation strategies, *Budorcas taxicolor bedfordi*

## Abstract

*Budorcas taxicolor bedfordi* is a rare animal uniquely distributed in the Qinling Mountains (China). Human disturbance and habitat fragmentation have directly affected the survival of *B. t. bedfordi*. It is urgent to clarify the genetic diversity and genetic structure of the *B. t. bedfordi* population and implement effective conservation measures. In this study, 20 new polymorphic microsatellite loci were isolated by Illumina sequencing. The genetic diversity and population structure of 124 *B. t. bedfordi* individuals from three populations (Niubeliang population, Zhouzhi population, and Foping population) were analysed according to these 20 microsatellite loci. Our results indicated that *B. t. bedfordi* had a low level of genetic variability and that there was inbreeding in the three populations. The population genetic structure analyses showed that the Niubeliang population had a trend of differentiation from other populations. National roads can affect population dispersal, while ecological corridors can promote population gene exchange. None of the three *B. t. bedfordi* populations experienced bottleneck effects. For conservation management plans, the Zhouzhi population and Foping population should be considered one management unit, and the Niubeliang population should be considered another management unit. We suggest building an ecological corridor to keep the habitat connected and formulating tourism management measures to reduce the influence of human disturbance on *B. t. bedfordi*.

**Supplementary Information:**

The online version contains supplementary material available at 10.1186/s40850-024-00192-1.

## Introduction

Biodiversity is the summation of all variations in life, and it is the material basis for human survival and development. With the growth of the population and the development of technology after the Industrial Revolution, human beings have become increasingly capable of utilizing and transforming nature. Human activities have seriously damaged the ecosystem by changing the climate or the environment or by encroaching on habitats. Forest habitat degradation and fragmentation have made a large number of species extinct or endangered [1–3]. The population size of many species is decreasing rapidly, and the genetic diversity within species is also declining [4]. Due to human activities, habitat fragmentation and other factors, many endangered species have low genetic diversity [5–9]. Consequently, there is an urgent need to study the genetic polymorphisms of each endangered species, especially those with very small distribution areas, to prevent the further decline of their populations. Determining how to repair the forest habitat environment and protect wild animals reasonably and effectively has become an urgent task for the survival and development of human beings.

Golden takin, *Budorcas taxicolor bedfordi*, belongs to the order Artiodactyla and family Bovidae. The forest habitat of this species is uniquely located in the Qinling Mountains, which covers a 24,773 km^2^ area between 32° N to 34° N and 106° E to 110° E with elevations of 1,550 m to 3,600 m above sea level in China [10–13]. The population quantity is 5500 ~ 8000 [8], and this species is a national first-class protected wild animal in China and is listed in CITES Appendix II [14]. The International Union for the Conservation of Nature and Natural Resources (IUCN) classified *B. t. bedfordi* as vulnerable (VU) [15]. The nature reserves in the distribution area of *B. t. bedfordi* are small and disconnected. Moreover, in the distribution area of *B. t. bedfordi*, there are 108, 210, 312, and 316 National Roads; 102 and 212 provincial roads; Xi ‘an to Hanzhong, Xi ‘an to Ankang, and Xi ‘an to Wuhan expressways; Baoji to Chengdu and Xi ‘an to Ankang railway special lines; and the recently built Xi ‘an to Chengdu high-speed railway. Although these roads connect the northern and southern regions of the Qinling Mountains and promote economic development, they divide the *B. t. bedfordi* habitat into multiple patches. Due to large-scale forest fragmentation and habitat fragmentation, the *B. t. bedfordi* populations in the Qinling Mountains are isolated from each other, and their living conditions are not optimal [16].

To formulate scientific and effective protection measures, the scientific evaluation of the population genetic diversity and genetic structure of *B. t. bedfordi* is necessary. Since microsatellite loci can provide high-resolution genetic information, they are very suitable for population genetic studies of wild animals such as *B. t. bedfordi* with a small population distribution area [17–18]. Compared with traditional microsatellite loci isolation methods, next-generation sequencing (NGS) techniques are more efficient and less costly [19, 20]. In this research, we isolated 20 microsatellite markers by NGS techniques and analysed the population genetic diversity and genetic structure of *B. t. bedfordi* in detail. The purposes of this research were as follows: (1) to examine the genetic variability of the species; (2) to appraise the population genetic structure; and (3) to analyse the influence of human disturbance on gene flow among populations to investigate the phylogeographical and landscape genetic patterns of *B. t. bedfordi*.

## Methods

### Tissue sample, DNA extraction and genome sequencing

**Fig. 1:**
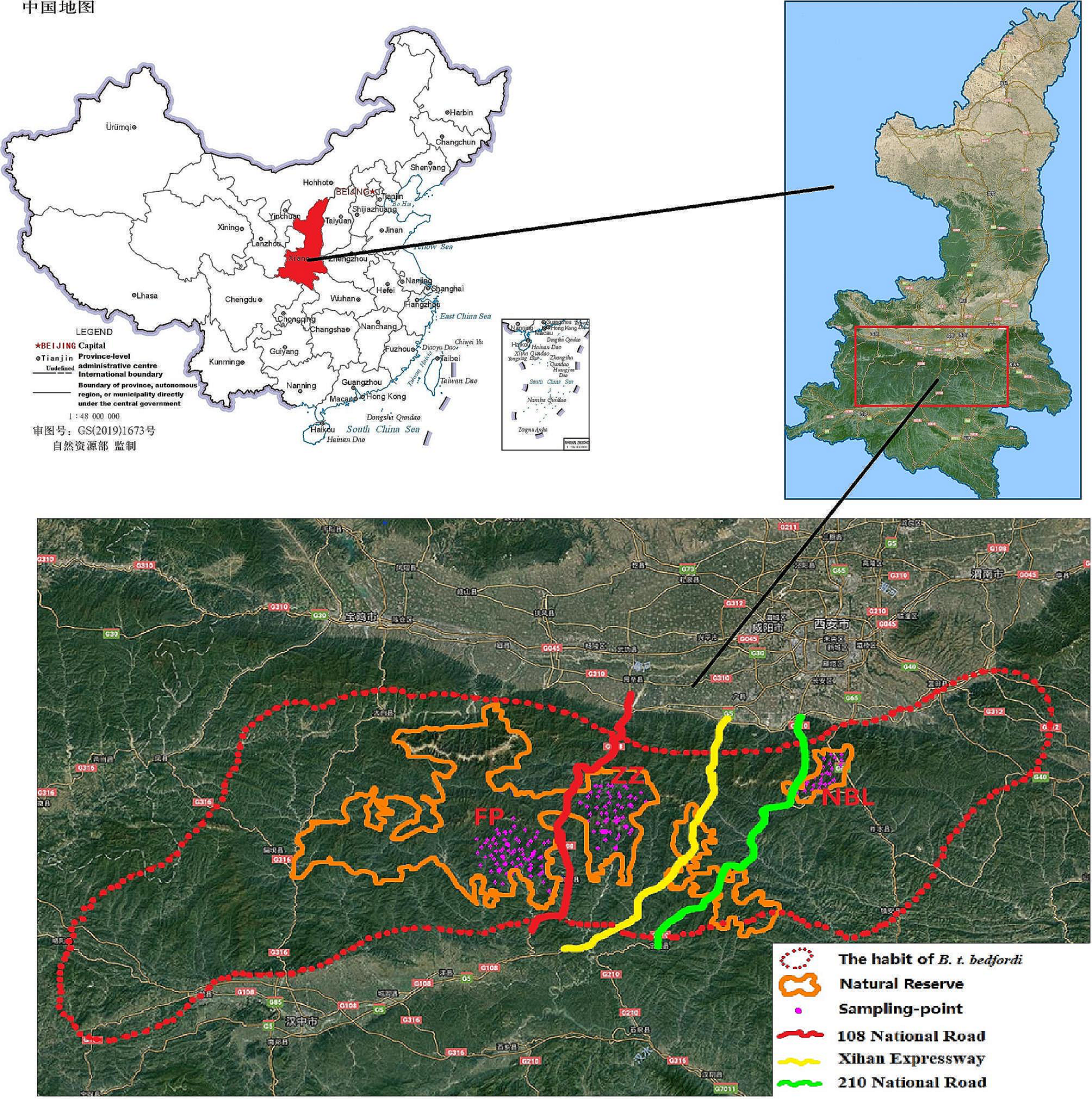
The habitat of *B. t. bedfordi* and distribution of samples. The map of China was based on the standard map service system GS (2019)1673 of the Ministry of Natural Resources (http://bzdt.ch.mnr.gov.cn/index.html) and the map of Shaanxi Province was based on Shaanxi Provincial Platform for Common GeoSpatial Information Services (http://shaanxi.tianditu.gov.cn. The base map has not been modified

Muscle samples (*n* = 124) were collected from dead *B. t. bedfordi* within three geographical regions: Zhouzhi National Nature Reserve (ZZ population, 108° 11′ E 33°45′ N; *n* = 40), Foping National Nature Reserve (FP population, 107° 53′ E 33°32′ N; *n* = 48) and Niubeiliang National Nature Reserve (NBL population, 109° 02′ E 33°91′ N; *n* = 36) (Fig. 1). The animals died of natural causes in the wild and were found by the reserve staff while they were patrolling the mountains. They had been dead for five to thirty days when they were found. The All muscle tissue samples collected from dead individuals were fixed in 95% ethanol. A QIAGEN DNeasy Blood & Tissue Kit (Qiagen, Valencia, CA) was used to extract whole genomic DNA [21].

Whole genomic DNA was enzyme digested into 400–500 bp fragments. The *B. t. bedfordi* DNA library was prepared by terminal repaired, adding poly A tail, ligating sequencing adapter, purification and PCR amplification. The purified DNA library was quantified using Agilent 2100 Bioanalyzer and ABI StepOnePlus Real-Time PCR System, and sequenced using Illumina NovaSeq 6000 platform.

### Microsatellite characterization and development

The mean quality score, Q20 values, Q30 values and the GC content of the sequencing raw data were estimated by seqtk 1.3 [22]. Microsatellite repeat units (di-, tri-, tetra-, penta-, hexanucleotide repeats) were scanned by Galaxy server pipeline [23, 24]. Primers were designed by Primer Premier 5.0 [25], using design parameters as follows: GC content between 50% and 70%, primer length range between 18 and 26 bp, target amplicon length range between 80 and 400 bp, annealing temperature between 55 and 66 °C. We designed three pairs of primer for each microsatellite locus and chose the primer pair with the highest score. From the novel microsatellite markers, we randomly selected 100 pairs of primers for further studies. The primers were synthesized by Beijing Tsingke Biotech Co., Ltd. (Beijing, China).

### Primer testing and selection

PCR amplification was carried out with 100 primer pairs using mixed DNA pool of golden takin. After agarose-gel electrophoresis, primer pairs that can amplify target bands were used as primary primers. The PCR amplification system was as follows: 1 μL *B. t. bedfordi* DNA, 12.5 μL PCR Master mix (TIANGEN, Beijing), 2 μL primer pairs, 9.5 μL ddH_2_O. The PCR reactions were as follows: initial denaturation at 94 °C for 8 min; 38 cycles of denaturation for 30 s at 94 °C, specified annealing temperature for 30s, and 72 °C for 45s; and 72 °C extension for 10 min. The primary primers were validated by direct sequencing and capillary electrophoresis (Beijing Tsingke Biotech Co., Ltd. Beijing, China).

**Table 1:** Information of twenty new microsatellite loci for *B. t. bedfordi*

Locus Name	Accession Number	Primer sequence (5′–3′)	Repeat motif	Ta (°C )	Size (bp)	N_A_	N_O_	H_O_	H_E_	P_HWE_	PIC
P1	MN733993	F:AATCAGATGGCCCTGAGGTA R:GCCACTGGCCTAAACCATT	(TG)24	61	237–284	8	0.1720	0.1742	0.7424	0.0587	0.593
P2	MN733994	F:AATTCCATGGACAAACGAGC R:CCTTGGAATTCAACCTTGTCA	(AC)30	59	142–201	14	0.2083	0.3185	0.5806	0.0132	0.851
P3	MN733995	F:AGACCTCGTGCAGTCGAATAA R:CCTTGGAATTCAACCTTGTCA	(AC)23	60	195–234	5	0.0021	0.7546	0.5511	0.2431	0.448
P4	MN733996	F:AGCTTGCACAAGCCATAGT R:GCATGGAATCCAGGGTAAGA	(TG)18	60	223–258	7	0.2694	0.2048	0.7259	0.0001	0.540
P5	MN733997	F:ATGAACATATCTGACCCGGC R:GCACTTGGGAAGATGGCTTA	(AC)22	60	205–248	11	0.0686	0.4801	0.7614	0.0013	0.646
P6	MN733998	F:CAAAGGCTGGGTGGTAAAG R:AAGAATCTGCCTGAAATGCG	(TG)19	61	274–311	11	0.2228	0.2559	0.7774	0.0037	0.557
P7	MN733999	F:CCTGGTATGCTCCAGTCCAT R:CCATTTCCAGCCAGGACTTA	(CA)22	59	238–281	11	0.1876	0.3608	0.7675	0.0021	0.462
P8	MN734000	F:CTTTGCAGGATGCTGTTGAA R:GCCACCTGAACTTACCCAAT	(GT)18	58	342–377	13	0.1852	0.3882	0.8504	0.0043	0.665
P9	MN734001	F:GCTTCAGGATCCAGCAGTTC R:CAGAGTCTGGCCTTCTGAC	(TG)23	60	401–446	9	0.2690	0.1152	0.8295	0.0026	0.736
P10	MN734002	F:TAACTTGGTCTGTTCCGGCT R:CTTCCCTACTGGCTCAGACG	(TG)18	59	232–267	12	0.1701	0.3675	0.8446	0.0031	0.772
P11	MN734003	F:TACTTCCCGAAACACCAAC R:TGTAGTGCCTGTCCAGGAG	(CA)19	60	428–465	6	0.0655	0.4415	0.7378	0.3526	0.477
P12	MN734004	F:TTATTTCAGGGCGGATCTTG R:ACCATAGGACAATGCGGAAG	(TG)21	59	243–284	13	0.2101	0.4468	0.8380	0.0002	0.629
P13	MN734005	F:AGGCTATGGTCCATGCAGTC R:AATACTTTGGCCACCTCACG	(AC)16	60	323–354	5	0.0013	0.1204	0.5954	0.0041	0.421
P14	MN734006	F:CTGCTAATCCCAAACTCCCA R:GCATCCCAATGTTCATAGCA	(GT)17	60	328–361	6	0.2036	0.5540	0.6504	0.0022	0.448
P15	OP970799	F:TTCCCTGAATCATTCCTTGG R:CCAGGCCATGTTTCTTCAAT	(TG)21	60	182–223	7	0.1751	0.3192	0.6933	0.114	0.565
P16	OP970800	F:CTGATCCTGCCCTGAAATGT R:ACTGGGTGCTGTAGTGCCTT	(AC)17	60	161–194	6	0.2129	0.4227	0.7323	0.0035	0.746
P17	OP970801	F:CTTCCTGAATGGGCTGGTTA R:TGAACAGCTTCAGCATCACC	(TG)17	60	289–322	12	0.1166	0.3615	0.8311	0.0010	0.601
P18	OP970802	F:GCTGTTTCCCATGTCCAGTT R:TGAAATAGGAAGCTCAGGGC	(AC)16	60	233–264	6	0.3154	0.2028	0.7098	0.0029	0.478
P19	OP970803	F:TGCAATGCAGGAGATCTGAG R:GAGATGGTGGAGTGCCCTAA	(AC)24	60	211–258	6	0.0011	0.5875	0.5160	0.4765	0.538
P20	OP970804	F:TTGTTCAGGGTGTGGTTTGA R:TCCTCGCGTCATCTAGTGTG	(CA)25	60	90–139	6	0.2799	0.2440	0.7213	0.0007	0.465

*Ta*, annealing temperature; *P*_*HWE*_, *p* value for Hardy–Weinberg equilibrium, Ho, He, Na, No

### Data analysis

CONVERT 1.3 [26] and GenALEx 6.5 software [27, 28] were used to convert the format of the genetic data. The null alleles were detected by Microchecker, and the null allele frequency (*N*_*O*_) was calculated by Cervus 3.0 software [29]. We also used Cervus 3.0 to estimate the polymorphic information content (*PIC*) of each microsatellite locus. Hardy–Weinberg equilibrium (*P*_*HWE*_) was calculated by Microchecker. GenALEx 6.5 [27]was used to analyse microsatellite loci polymorphisms and population genetic diversity, including the number of different alleles (*N*_*A*_), number of effective alleles (*N*_*E*_), Shannon’s information index (*I*), expected heterozygosity (*H*_*E*_), observed heterozygosity (*H*_*O*_), and Wright’s inbreeding coefficient (*F*_*IS*_). Nei’s genetic distance among individuals was calculated by PHYLIP 3.6 [30]. The principal coordinates of three *B. t. bedfordi* populations were evaluated by GenALEx 6.5. INEST 2.0 [31] was used to estimate *F*_*IS*_ per population and the parameter setting was the default Bayesian approach. We used GENEPOP V4 [32] to estimate the pairwise population genetic differentiation index (*F*_*ST*_). The individual and population UPGMA trees were constructed by MEGA 5.2 [33, 34].

In addition, the genetic structure and the relationship among the three populations of *B. t. bedfordi* were analysed by using Structure 2.3.1 software [35]. The parameters were set as follows: number of populations (K), 1 to 10; length of burn-in period, 20000; and number of MCMC repeats after burn-in, 100000. The most likely value of the genetic Cluster K value is selected according to the principle of maximum likelihood value or calculation of the inflection point of the ΔK peak value.

Furthermore, we used two statistical methods to examine potential bottlenecks, heterozygote excess of populations and the mode-shift indicator test. Heterozygote excess was detected by Wilcoxon tests (one tail) using BOTTLENECK 1.2.02 software [36]. Wilcoxon tests were calculated under infinite allele (IAM), stepwise mutation (SMM), and two-phase (TPM) models, and each model was performed with 10000 simulation repeats. In the mode-shift indicator test, rare allele frequency will decrease if the population experiences a bottleneck effect. Consequently, when the allele frequency distribution follows the normal L-shaped form, it indicates that the population has not experienced a bottleneck effect.

To determine whether the isolation between populations was caused by distance factors or human interference, we calculated the correlation between genetic distance (*F*_*ST*_ among populations) and geographic distance of samples with the Mantel test of GenALEx [27]. The geographical distance between each sampling point was obtained by ArcGIS 10.5.

## Results

### Microsatellite detection and test of neutrality

A total of 374,645 microsatellite motifs were identified. Among these motifs, there were 259,891 (69.37%) dinucleotide repeats, 60,468 (16.14%) trinucleotide motifs, 46,681 (12.46%) tetranucleotide motifs, 4,983 (1.33%) pentanucleotide motifs and 2,622 (0.7%) hexanucleotide motifs. As a result, 20 microsatellite loci (GenBank accession numbers MN733993-MN734006 and OP970799-OP970804) with high polymorphism were isolated.

The value of *N*_*A*_ ranged from 5 to 14, the value of PIC ranged from 0.421 to 0.851, the value of average *H*_*O*_ was 0.3260, and the value of average *H*_*E*_ was 0.7360 (Table 1). After Bonferroni correction, there were thirteen loci (65%) that significantly deviated from Hardy-Weinberg equilibrium and seven loci (35%) that conformed to Hardy-Weinberg equilibrium (Table 1).

### Genetic diversity

In view of its small geographical distribution area and habitat fragmentation, we estimated the genetic diversity level of the *B. t. bedfordi* population to be low. The results of *B. t. bedfordi* genetic diversity from the three populations are shown in Table 2. The values of *I*, *H*_*E*_ and *UH*_*E*_ were lowest in the NBL population (*I* = 1.402 ± 0.083, *H*_*E*_ = 0.704 ± 0.029, *UH*_*E*_ = 0.714 ± 0.029). The values of the inbreeding coefficient over polymorphic loci among populations ranged from 0.462 ± 0.080 to 0.568 ± 0.091.

**Table 2:** Genetic diversity of three *B. t. bedfordi* populations (mean ± SE)

Population	N	N_A_	N_E_	I	H_O_	H_E_	UH_E_	F_IS_
ZZ	48	5.850 ± 0.477	4.616 ± 0.427	1.558 ± 0.085	0.309 ± 0.052	0.747 ± 0.023	0.756 ± 0.023	0.560 ± 0.084
FP	40	7.450 ± 0.564	4.687 ± 0.391	1.647 ± 0.079	0.314 ± 0.058	0.758 ± 0.019	0.766 ± 0.020	0.568 ± 0.091
NBL	36	5.050 ± 0.400	3.882 ± 0.298	1.402 ± 0.083	0.356 ± 0.047	0.704 ± 0.029	0.714 ± 0.029	0.462 ± 0.080

*N*, number of samples;*UH*_*E*_, unbiased expected heterozygosity

### Genetic structure

The results of the *F*_*ST*_ values among the three populations of *B. t. bedfordi* show that the *F*_*ST*_ value was lowest between the ZZ and FP populations (*F*_*ST*_ =0.026) and highest between the FP and NBL populations (*F*_*ST*_ =0.051) (Table 3). The results demonstrated that the NBL population had higher genetic differentiation than the ZZ and FP populations.

**Table 3:** Values of *F*_*ST*_ among three populations of *B. t. bedfordi*

Population	ZZ	FP	NBL
ZZ	0.000		
FP	0.026	0.000	
NBL	0.048	0.051	0.000

**Fig. 2:**
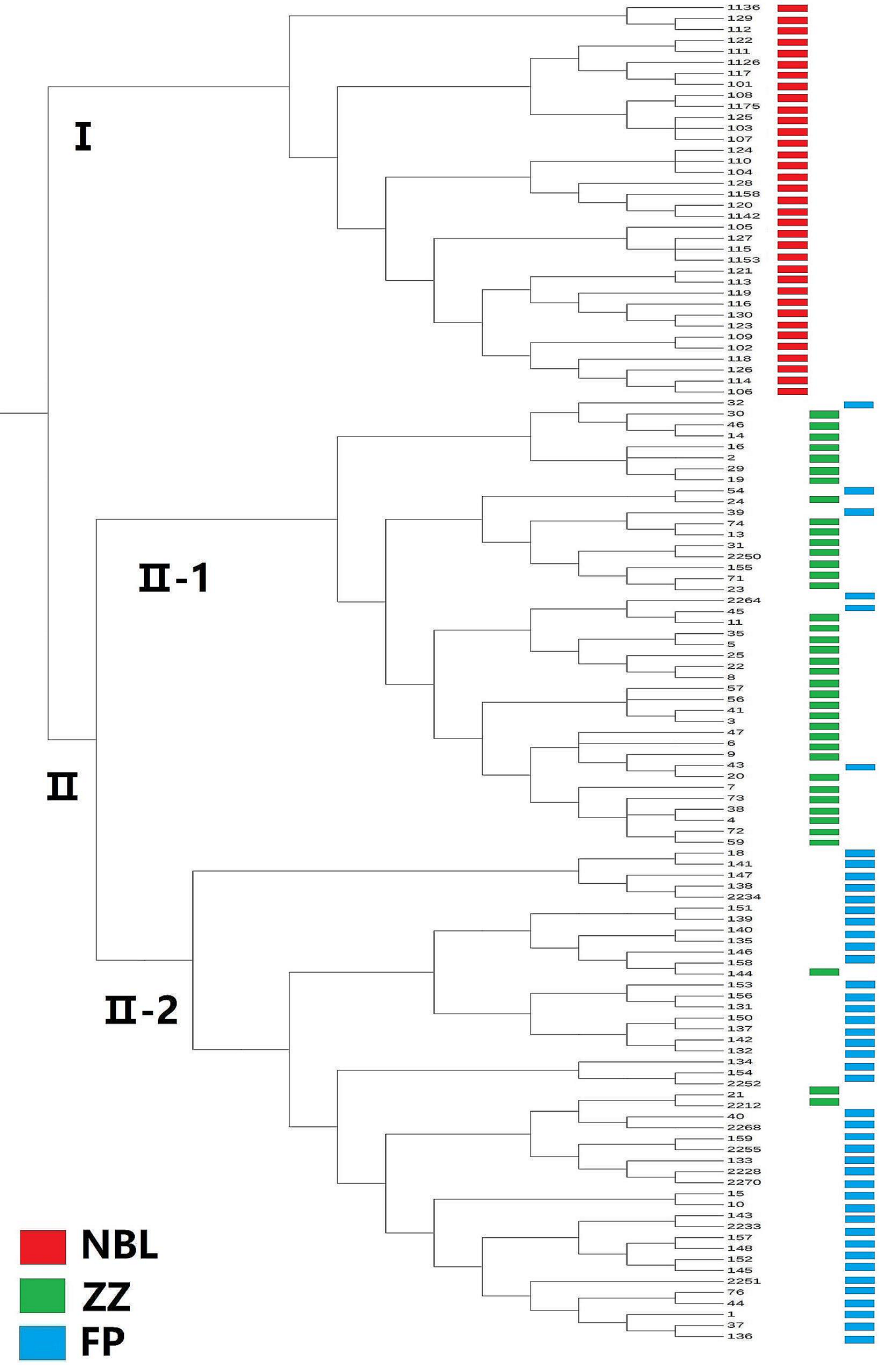
UPGMA phylogenetic tree of 124 individuals of *B. t. bedfordi*

The UPGMA phylogenetic tree of 124 individuals is shown in Fig. 2. The 124 individuals formed two large clades, clade I and clade II, of which clade II can be further divided into two small clades, clade II-1 and clade II-2.

Using different colours to label 124 individuals based on the collected regions, it was found that individuals from the NBL population were all clustered in clade (I) The individuals from the ZZ population and FP population were all clustered in clade (II) The ZZ population mostly clustered in clade II-1, and 3 individuals were distributed in clade II-2. Most of the FP population was clustered in clade II-2, and 6 individuals were distributed in clade II-1.

**Fig. 3:**
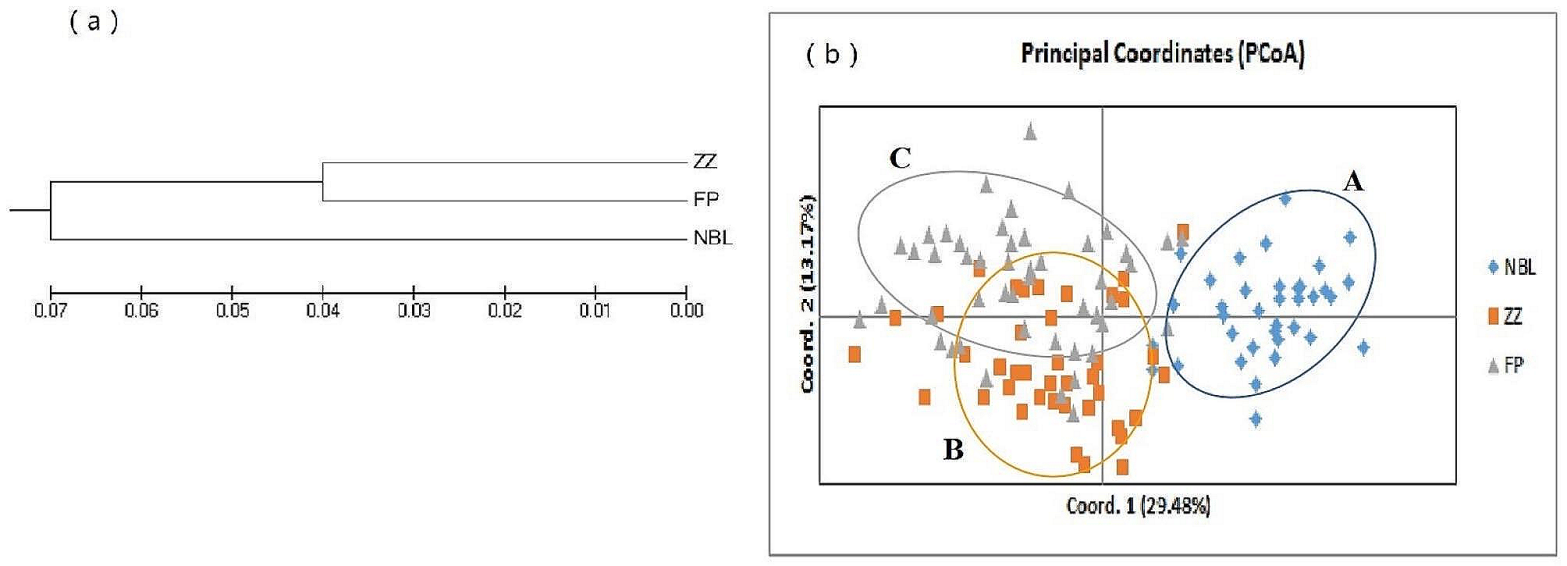
(**a**) UPGMA phylogenetic tree of three *B. t. bedfordi* populations; (**b**) principal coordinates of three *B. t. bedfordi* populations

A UPGMA phylogenetic tree of the three populations of *B. t. bedfordi* is shown in Fig. 3 (a). The three populations first gathered into two large branches. The ZZ population and FP population had a closer genetic relationship. The NBL population had the farthest genetic relationship with other populations.

The principal coordinate analysis showed that 124 individuals could be divided into three groups (Fig. 3(b)), and the clustering results were basically consistent with the phylogenetic tree. The NBL population was gathered in Group A, the ZZ population was mostly gathered in Group B, and the FP population was mostly gathered in Group C. The ZZ population and FP population had sporadic individuals distributed in Group C and Group B.

**Fig. 4:**
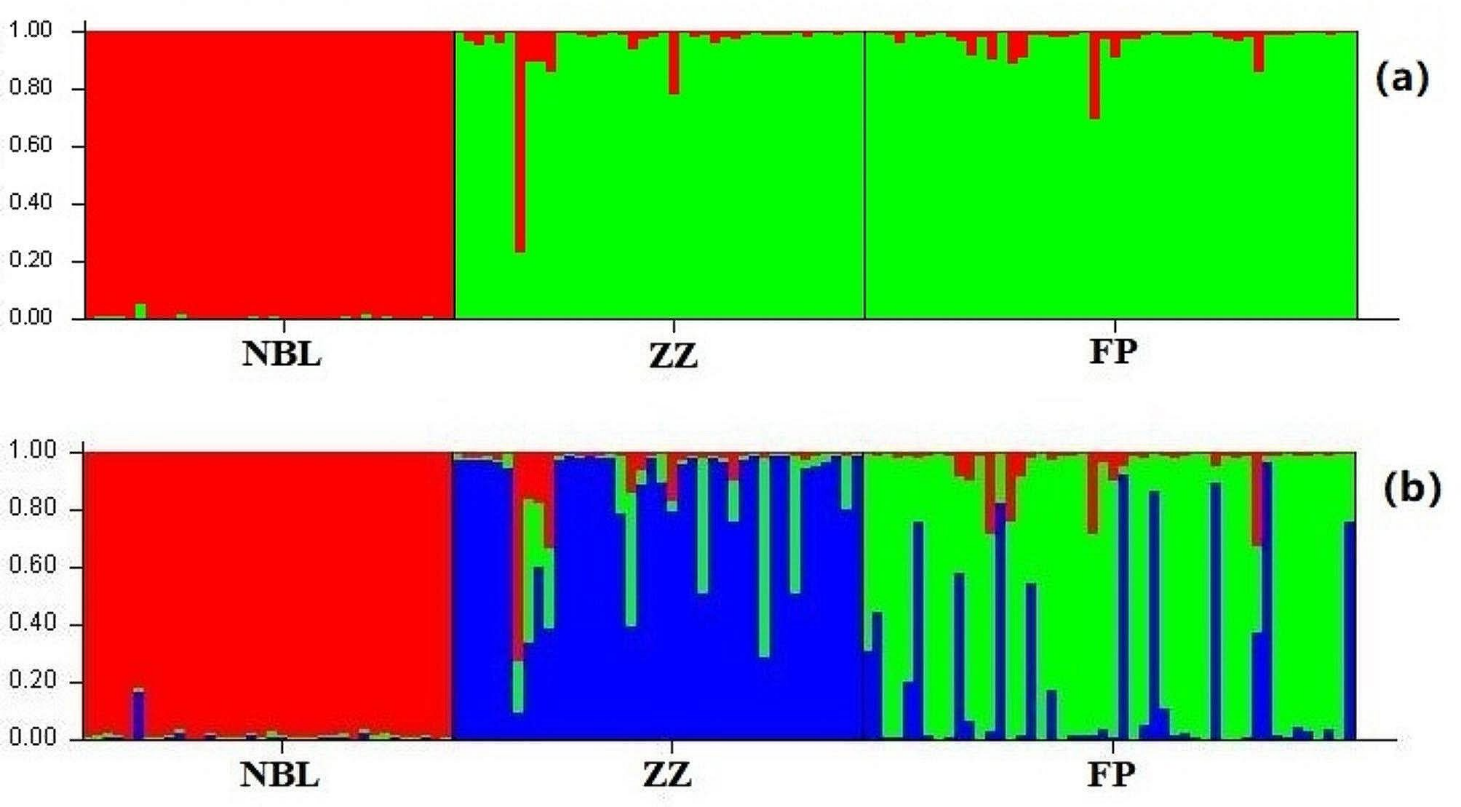
STRUCTURE bar plots for three populations of *B. t. bedfordi*. (a) K = 2, (b) K = 3

It can be seen from the population genetic structure analysis that when K = 2, ΔK had the maximum value (Supplementary Figure S1). All individuals from the Niubeiliang region were assigned to the red cluster, and all individuals from the Zhouzhi and Foping regions were assigned to another cluster (green) (Fig. 4 (a)). When K = 3, the green cluster was divided into two clusters (blue and green) (Fig. 4(b)). Except for the red cluster, the blue and green clusters were mixed with other colours, indicating that there was genetic exchange between the FP and ZZ populations.

### Test for bottleneck effects

Under the three assumptions of the IAM, TPM and SMM models, no significant heterozygosity excess was detected in any of the three populations (*p* > 0.05) and displayed a lack of heterozygosity. The results of the mode-shift indicator test are shown in Supplementary Table S1 and Figure S2. The values of allele frequency ranged from 0.1 to 0.7 and were mainly distributed at 0.1. Each population was characterized by an L-shaped distribution of allelic frequencies (i.e., many alleles with low frequencies). The results indicated that the *B. t. bedfordi* populations did not experience bottleneck effects.

**Table 4:** Test of bottleneck effects for *B. t. bedfordi*

Population	IAM (*p* value)	SMM (*p* value)	TPM (*p* value)
ZZ	0.89957	0.99999	1.00000
FP	1.00000	0.87996	0.72706
NBL	1.00000	0.92957	1.00000

## Discussion

### Genetic variation of ***B. t. bedfordi***

*PIC* is a commonly used indicator in genetic diversity research and is used to evaluate the identification ability of microsatellite markers and reflect the reliability of information provided by microsatellite loci [37, 38]. When *PIC* > 0.50, the polymorphism of microsatellite markers was higher, and the information provided was rich, which could well reflect the genetic diversity. When 0.25 < *PIC* < 0.50, microsatellite markers had high polymorphism and information content, which can provide more reasonable information. When *PIC* < 0.25, microsatellite markers had low polymorphism and provided less information [39]. All twenty microsatellite loci in this study had high *PIC* values, greater than 0.25, and the highest value was 0.851. Therefore, these 20 microsatellite markers were highly informative and were sufficient for discriminating among *B. t. bedfordi* individuals and populations.

Genetic diversity is essential because it is the raw material for natural selection allowing species to adapt to new environmental conditions. The loss of genetic diversity decreases species adaptive potentials [39]. The allele number and heterozygosity value are important indicators of population genetic diversity. The higher the values are, the higher the genetic polymorphism of the populations. We observed a total of 174 alleles from 20 microsatellite loci in this study, with an average number of 8.7 alleles. The number of effective alleles in the three sampling populations was 4.395, slightly lower than that of other endangered wild animals in the same region, giant panda (*Ailuropoda melanoleuca*) 4.58 [40], golden monkey (*Rhinopithecus roxellana*) 5.05 [41], forest musk deer (*Moschus berezovskii*) 6.37 [42], goat (*Capra hircus*) 5.23 [43], and cattle (*Bos taurus*)6.21 [44]. The average observed heterozygosity (*H*_*O*_) of *B. t. bedfordi* was 0.326, which was lower than that of the giant panda (0.488) [45], golden monkey (0.620) [46], goat (0.66) [47] and cattle (0.74) [44]. Our results confirm that the genetic variety of *B. t. bedfordi* was quite low. In addition, the genetic diversity of the *B. t. bedfordi* population was also studied based on the mtDNA D-loop region and single nucleotide polymorphisms (SNPs). The nucleotide diversity of *B. t. bedfordi* analysed by the mtDNA D-loop region was 0.0021 [48], indicating low genetic diversity [49]. The single nucleotide variants of *B. t. bedfordi* analysed by SNP were lower than those of the giant panda, golden monkey, goat, and cattle [50–52]. These results indicated that the *B. t. bedfordi* population suffered from poor genetic diversity, which was in accordance with the results of this study. At present, the number of golden takins in the Qinling Mountains is only approximately 5000 [53]. In approximately 1960, the quantity of *B. t. bedford*i was approximately 600. Later, the Chinese government implemented relevant protection policies and established protected areas, which gradually increased the quantity of *B. t. bedfordi*. However, the protected areas were not connected, and human disturbances were severe, which hindered communication between individuals from different regions. Therefore, although the population has increased in recent decades, the genetic diversity is low, and the inbreeding coefficient within the population is high. The low genetic diversity and small population size of *B. t. bedfordi* could have led to its near extinction. Consequently, there is an urgent need to protect *B. t. bedfordi*.

In the Hardy-Weinberg equilibrium test, 65% of the loci were out of equilibrium, and the three populations also deviated significantly from Hardy-Weinberg equilibrium. The three assumptions of IAM, TPM and SMM by Bottleneck showed that three populations of *B. t. bedfordi* presented a deficit of heterozygotes. This result indicated that the deviation of the *B. t. bedfordi* population from Hardy-Weinberg equilibrium was caused by heterozygote deficiency. Heterozygous deficiency is mainly caused by null alleles, inbreeding, and the Wahlund effect [54, 55]. In population genetics studies, null allele frequencies below 0.2 did not affect the result analysis [56, 57]. In this study, out of 20 loci, only 1 locus had a null allele frequency of 0.31, thus indicating that the null allele did not affect the accuracy of the analysis in this study [58]. In addition, the *F*_*IS*_ of the three groups were all greater than 0, and the average *F*_*IS*_ value reached 0.53, indicating that there was significant inbreeding among the *B. t. bedfordi* populations [59]. Furthermore, it can be seen from the results of the *F*_*ST*_ values, phylogenetic analyses, and population genetic structure analysis that *B. t. bedfordi* had significant population structure (Table 4; Figs. 3 and 4). The significant population structure can lead to a potential Wahlund effect [60, 61]. Consequently, we suspected that heterozygote deficiency was mainly due to inbreeding or population substructure. Although the *B. t. bedfordi* population did not experience a bottleneck effect and the population increased year by year, the high inbreeding coefficient and heterozygote deficiency will cause *B. t. bedfordi* to easily suffer from the bottleneck effect in the case of abrupt habitat change.

### Effects of forest change on the population genetic structure of ***B. t. bedfordi***

The genetic differentiation among the *B. t. bedfordi* populations was low. However, the *F*_*ST*_ value of the NBL population and the FP population reached 0.051, indicating that the NBL population had a trend of differentiation. At the same time, the Structure results also showed that all *B. t. bedfordi* individuals were divided into two groups, and the NBL population was a separate group. It can also be seen from the Structure results that when K = 3, there is a certain differentiation between the FP and ZZ populations. We examined the correlation between genetic distance and geographical distance and found that the correlation was not significant (*R* = 0.358, *P* = 0.480) (Figure S3), suggesting that the genetic differences between *B. t. bedfordi* populations were incompletely caused by distance. As seen from the satellite map, the three *B. t. bedfordi* populations were separated from each other by 108 National Roads and 210 National Roads (Fig. 1). On both sides of the road, the vegetation was seriously damaged, and the crown density decreased, which seriously affected the population dispersal of *B. t. bedfordi.* 108 National Road, built in 1969, obstructed individual communication between the ZZ population and FP population in the short term. In addition, in 2000, the Qinling Tunnel of 108 National Road was put into use, while the mountaintop section was abandoned. Vegetation restoration based on forest care and renewal and planting of native tree species was carried out at the same time. Therefore, the gene flow with the ZZ population and FP population was facilitated, and the genetic differentiation was small. 210 National Road, built in 1930, blocked individuals of the NBL population for a long time. Moreover, in 2002, the Xihan Expressway was built between the habitats of the NBL and ZZ populations, which increased the difficulty of individual communication between the NBL population and the other two populations. These two roads also produced certain barriers to the gene flow of golden monkey populations [46]. Thus, it can be inferred that the genetic differentiation between *B. t. bedfordi* populations was caused not only by distance but also by the influence of roads.

### Conservation for ***B. t. bedfordi***

The current results indicated that *B. t. bedfordi* had low genetic diversity and inbreeding within the population, and its distribution range was small. The species is vulnerable to abrupt habitat change or infectious disease and faces an uncertain future. Thus, we propose the following protection recommendations.

1. The gene flow between the ZZ population and FP population was facilitated, so *B. t. bedfordi* inhabiting these two reserves can be protected as a whole population. The NBL population can be protected as a separate unit. In addition, it was found that national roads and expressways may interfere with the migration of *B. t. bedfordi*, blocking its gene flow. We suggest building ecological corridors in the meadow area of the NBL reserve to reduce or remove the impact of habitat fragmentation on the migration of *B. t. bedfordi* and gradually improve the quality of the habitat of *B. t. bedfordi.* Therefore, the habitat available for *B. t. bedfordi* can be connected and intact, and genes can be exchanged freely [62].
2. Because there is a small number of *B. t. bedfordi* distributed in the surrounding areas of each reserve, we propose that the surrounding areas should be designated as the extension range of the reserve and the habitat range should be gradually expanded to satisfy the population increase.
3. We suggest that the tourist administration bureau and relevant departments scientifically plan the norms of forest parks, the length of time scenic spots are open and control the size of tourist flow [63, 64]. These measures can reduce the interference with the daily activities and migration of *B. t. bedfordi* caused by roads and tourists as much as possible.
4. It is necessary to carry out ex situ conservation. The rescued *B. t. bedfordi* can be released in different places to support the genetic exchange with different populations.

## Conclusion

In conclusion, the results obtained in this study indicate that the genetic diversity of *B. t. bedfordi*, an endangered wild animal, is quite low. Due to forest fragmentation and road construction, genetic differentiation among populations has occurred. However, ecological corridor construction and forest restoration were beneficial to individual exchange. We have proposed some measures for the conservation of *B. t. bedfordi* population diversity in the Qinling Mountains. This study provides a solid foundation for further research on the endangerment mechanism of *B. t. bedfordi*.

### Electronic supplementary material

Below is the link to the electronic supplementary material.

**Supplementary Material 1: Figure S1** Criteria for selecting the optimal K for assigning individuals during the Bayesian cluster analysis. **Table S1**. The proportion of alleles in different allelic frequency classes for different populations of *B. t. bedfordi*. **Figure S2**. Graphic representation of proportion of alleles in different allelic frequency classes for different populations of *B. t. bedfordi*. **Figure S3**. Relationship between pairwise FST/(1-FST) and geographic distance among populations of *B. t. bedfordi*

## Data Availability

The datasets generated and analysed during the current study are available in the KNB repository, https://knb.ecoinformatics.org/view/doi:10.5063/F1ST7N9D.
